# Birch rust allergy as a novel autumnal trigger of seasonal airway symptoms

**DOI:** 10.1016/j.jacig.2025.100599

**Published:** 2025-11-06

**Authors:** Randi Falnes Olsen, Elin Ørbeck Vallevik, Thorsten Graf, Kristian Svendsen, Annette Kuehn, Martin Sørensen

**Affiliations:** a Regional Center for Asthma, Allergy and Intolerance, University Hospital of North Norway, Tromsø, Norway; b Department of Paediatric and Adolescent Medicine, Innlandet Hospital Trust, Lillehammer, Norway; c Department of Infection and Immunity, Luxembourg Institute of Health, Strassen, Luxembourg; d Department of Pharmacy, the Arctic University of Norway, Tromsø, Norway

**Keywords:** Airway allergy, birch rust fungi, seasonal allergy

## Abstract

**Background:**

Accurate identification of allergenic triggers is essential for diagnosis, prevention, and management of airway allergies. In Tromsø (in northern Norway), birch rust (BR) spores are released in large quantities during autumn, a period with minimal dispersal of other environmental aeroallergens. This seasonal spike in BR spores coincides with an increase in symptoms of conjunctivitis, rhinitis, and asthma.

**Objective:**

Our aim was to investigate whether exposure to BR spores is associated with allergic airway symptoms in autumn.

**Methods:**

A total of 31 patients and 17 controls residing in Tromsø used a visual analog score to record their daily airway allergy symptoms from August 4 to October 14, 2020. Levels of specific IgE against BR and other relevant aeroallergens were measured in 13 patients and 7 controls. Skin prick testing for BR was conducted in 5 patients and 2 controls. Because of the lack of commercial reagents, test extracts from sampled BR spores were developed and produced during the study period. Airborne spore and pollen counts were collected from a monitoring station in Tromsø.

**Results:**

The mean symptom visual analog scale scores were significantly higher in the patients than in the controls. Of the 13 patients tested, 10 (76.9%) demonstrated specific IgE reactivity to BR spores. All 5 patients undergoing skin prick testing showed positive reactions to BR extracts.

**Conclusions:**

These findings suggest that BR spores may represent previously unrecognized aeroallergens contributing to seasonal allergic airway symptoms in autumn. Further studies are needed to validate these preliminary observations and to clarify the underlying nature of this allergy.

## Introduction

Allergic airway diseases, such as allergic rhinitis, conjunctivitis, and asthma, affect more than 30 % of the global population,^1^, ^2^, ^3^ causing substantial burdens for both patients and society.^2^^,^^4^^,^^5^ These conditions often coexist and are triggered by specific allergens, making the identification of the causative allergen(s) essential for accurate diagnosis as well as for guiding prevention and treatment strategies.^6^^,^^7^

Fungi are widespread in the environment, and several species are known contributors to allergic airway diseases (eg, the outdoor molds *Alternaria alternata* and *Cladosporium herbarum*).^8^ Seasonal allergic symptoms typically arise when fungal spore concentrations in the outdoor air are high,^9^ as seen from June to October in central Europe. In northern Norway, however, the specific triggers of allergic airway symptoms in autumn remain poorly understood.

**Fig 1:**
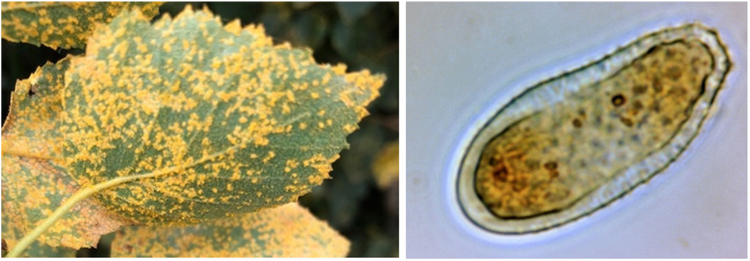
Photographs of BR on leaves (courtesy of Martin Sørensen) and BR spore (courtesy of the Luxemburg Institute of Health).

The region of Tromsø in northern Norway has a subarctic climate, in which tree pollen spread in May to June whereas grass pollen and *Cladosporium* spores spread in July to August.^10^ Concentrations of *Cladosporium* spores are usually below the reported clinical thresholds, with annual mean air concentrations of 413 spores/m^3^ (2004-2023).^11^ Since 2004, environmental monitoring stations in Norway have also acquired spore counts for the emerging fungus *Melampsoridium betulinum,* which is commonly referred to as birch rust (BR). This fungus affects the leaves of birch trees as its primary host, appearing as yellow spots on birch leaves (Fig 1). BR is prevalent in birch forests across the cool temperate and boreal regions of the Northern Hemisphere (north of the 50th parallel), particularly in high-elevation areas,^11^^,^^12^ with seasonal sporulation and spore dispersal occuring in the fall.^8^ In the region of Tromsø, BR spores spread from mid-August until early October, with annual mean air concentrations of 1504 spores/m^3^ (2004-2023).^11^

We hypothesized that allergic airway symptoms occurring in autumn, when there is no spread of other known aeroallergens, are caused by BR spores as a novel allergy trigger.

## Results and discussion

Seasonal allergic airway symptoms occur in response to exposure to pollen from wind-pollinated plants and spores from mold.^10^^,^^13^, ^14^, ^15^ Although more than 100 fungal proteins have received official allergen names to date (https://allergen.org/; data retrieved on October 6, 2025), documentation of allergenic properties remains limited for most fungi. So far, there are no published data on allergic sensitization to BR spores, and only isolated reports suggest that other rust fungi may act as allergenic sources.^16^^,^^17^ In this pilot study, we aimed to investigate whether there is an association between exposure to BR spores and allergic airway symptoms in autumn, during a period when other aeroallergen levels are minimal.

We recruited 31 participants with clinical symptoms of allergic airway disease during BR spore season (hereafter referred to as patients*)* and compared them with 17 participants without such symptoms or any known history of allergy (hereafter referred to as controls*)*. Age and sex were distributed similarly between groups (Table I), whereas the patients exhibited a more pronounced atopic profile. Most patients had a history of allergic conditions, including asthma, rhinitis, and eczema, reflecting the well-established comorbidity among atopic diseases.^7^ The frequent overlap between asthma and rhinitis appeared particularly relevant in this cohort. More than 70% of patients reported allergic reactions to pollen: 38.7% to birch pollen, 45.2% to grass pollen, and 13.9% to both, which is substantially higher than the estimated 20% to 25% prevalence in the general Norwegian population,^18^ highlighting the strong atopic burden in the patient group. In contrast, only 1 control (5.9 %) reported pollen allergy.

**Table I:** Demographics of the pilot cohort (N = 48) comparing the group with clinical symptoms and the group without clinical symptoms

General characteristic	Patients (n = 31 [64.6%])	Controls (n = 17 [35.4%])	*P* value
Age group (y)			
18-25	3 (9.7%)	0 (0%)	.54
26-35	5 (16.1%)	3 (17.4%)	1.00
36-45	5 (16.1%)	6 (35.3%)	.13
46-55	10 (32.3%)	2 (11.8%)	.17
56-65	2 (6.5%)	3 (17.4%)	.33
≥66	6 (19.4%)	3 (17.4%)	1.00
Female sex	20 (64.5%)	12 (70.6%)	.67
Symptom experienced			
During past 12 mo∗			
Asthma	16 (51.6%)	0 (0%)	**<.001**
Allergic rhinitis	18 (58.1%)	0 (0%)	**<.001**
Eczema	11 (35.5%)	0 (0%)	**<.01**
Pollen allergy	22 (71.0%)	1 (5.9%)	**<.001**
HDM allergy	7 (22.6%)	0 (0%)	.04
Pet allergy	16 (51.6%)	0 (0%)	**<.001**∗
Ever†			
Asthma	17 (54.8%)	0 (0%)	**<.001**∗
Allergic rhinitis	19 (61.3%)	2 (11.8%)	**.01**∗
Eczema	14 (45.2%)	4 (23.5%)	.21
Familial atopic disease†	24 (77.4%)	9 (52.9%)	.08
During BR spore season‡ (mean visual analog scale score)§			
Total allergic symptoms	2.42 (1.23-3.58)	0 (0-0)	**<.001**∗
Allergic rhinitis	2.52 (1.00-3.10)	0 (0-0.05)	**<.001**∗
Allergic conjunctivitis	1.08 (0.23-2.65)	0 (0-0)	**<.001**∗
Allergic asthma	0.10 (0-1.50)	0 (0-0)	**<.001**∗

*P* values were calculated using the chi-square or Fisher exact test for categoric values and Student *t* test for continuous values, followed by the Benjamini-Hochberg procedure; α = 0.05. Boldface indicates statistical significance.

∗ Based on questions from the MeDALL (Mechanisms for the Development of Allergies) questionnaire, as detailed in the Supplementary Methods section (in the Online Repository at www.jaci-global.org).

† Self-reported, doctor-diagnosed allergy; familial atopic disease (in parents, siblings, children, and/or grandchildren).

‡ In all, there were 30 patients and 17 controls with symptom registration.

§ Based on visual analog scale–rated symptom registration, as detailed in the Supplementary Methods.

**Fig 2:**
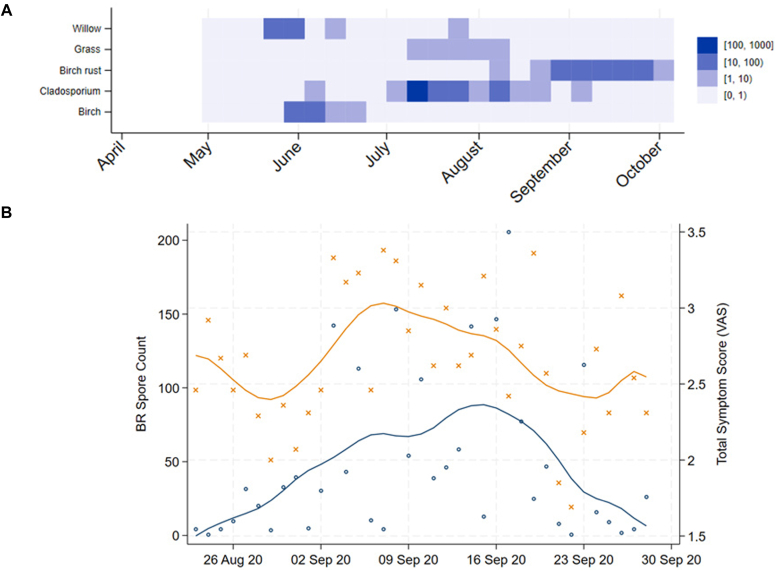
**A,** Weekly concentrations of tree (willow, birch), grass, *Cladosporium*, and BR (*Melampsoridium betulinum*) spores in Tromsø from April to October 2020. *Alternaria* and mugwort were not detected. **B,** Mean total allergic symptom scores (visual analog scale [VAS] [*orange*]) in patients and daily BR spore counts (*blue*) during the dispersal season (August 23-Sepember 28).

Relevant pollen and spore seasons in Tromsø in 2020, as obtained by using data from the sole monitoring station in the region, are shown in Fig 2, *A*. BR spore dispersal lasted from early August to early October, with a total count of spores in the air of 1858/m^3^, which surpassed the annual mean concentration of 1504 spores/m^3^.^11^ Nearly all spread of *Cladosporium* (80%) occurred before the BR season started, and there was almost no spread of *Alternaria*. Birch pollen dispersal took place in June, followed by grass pollen dispersal in July to mid-August, with total pollen counts of 281 and 145 pollen grains/m^3^ of air, respectively.^11^ These data from the national registration of pollen and spores in Tromsø confirm that only minimal concurrent spread of other aeroallergens occurred during BR season.^10^^,^^11^ With a high BR spore dispersal and limited interference from other respiratory allergens, Tromsø provides a unique environment to study BR spores as airway allergens.

As an inclusion criterion, patients were selected on the basis of a history of seasonal allergic rhinitis, conjunctivitis, and/or allergic asthma occurring annually from August to October. To ensure a clear clinical contrast and minimize the risk of symptom overlap or undetected BR sensitization, a group of allergy-free individuals was recruited as controls. All participants recorded their allergic symptoms daily during the BR season (see Symptom Registration Questionnaire in Online Repository Methods). Symptoms were categorized as total allergic symptoms, rhinitis, conjunctivitis, or asthma, and severity was rated by using a visual analog scale. Compared with the controls, the patients reported significantly more symptoms across all symptom categories during the BR spore season (*P* < .001 [see Table I]). Among the patients with symptom registration (n = 30), a positive but nonsignificant association between symptom severity and BR spore levels was observed in a mixed-model, multilevel linear regression (*P* = .079). This temporal trend, which is illustrated in Fig 2, *B*, supports a potential association between BR exposure and onset of allergic airway symptoms.

Serum-specific IgE to common aeroallergens was measured in participants residing near the pollen trap (13 patients and 7 controls) to assess whether other sensitizations could explain the symptoms reported during the BR season (Table II). Approximately 60% of the patients were sensitized to pollen and/or animal dander, whereas only 1 showed low-level sensitization to house dust mite (HDM). No sensitization to other fungal allergens was detected among the patients, and none of the controls showed sensitization to any of the tested aeroallergens. These findings, together with self-reported histories of atopic disease, suggest that individuals with preexisting atopy may be more susceptible to sensitization to BR. However, these coexisting sensitizations do not explain the observed symptoms, as the BR season occurs after the peak pollen season. Furthermore, sensitization to pets or HDM is unlikely to account for these reactions, as fewer than half of the patients were sensitized to animal dander and only 1 was sensitized to HDM.

**Table II:** s-IgE to common aeroallergens, as measured for 13 patients

Patient	Total IgE level	Cat	Horse	Dog	Rabbit	*D pter*	Grass	Birch
1	36.0	0	0	0	0	0	0	0
2	360.0	0	0	0	0	0	0.1	85.3
3	513.0	0	0	0	0	0	0	0
4	279.0	0	0	0	0	0	8.8	0
5	34.2	0	0	0	0	0	0	0
6	32.9	3.2	0.6	1.4	0	0	6.9	0
7	204.0	13.6	4.5	1.8	0	0	13.2	18.2
8	34.4	0.8	0	0	0	0	0	0
9	151.0	2.3	1.5	0.8	2.1	0	41.8	0.5
10	115	2.0	1.0	15.9	0	0	0	4.5
11	127.0	0.7	0	1.4	0	0.5	19.2	3.9
12	43.9	0	0	0	0	0	0.4	1.0
13	42.0	0	0	0	0	0	8.2	0

*D pter, Dermatophagoidespteronyssinus*.

Birch refers to birch pollen, and timothy refers to grass pollen.

The numeral 0 indicates an s-IgE level less than 0.35 kU/L. No patients were sensitized to mugwort, *A alternata*, *C herbarum*, *Penicillium notatum*, *Aspergillus fumigatus,* or *Mucor racemosus*. None of 5 controls were sensitized to any of the aeroallergens measured.

**Fig 3:**
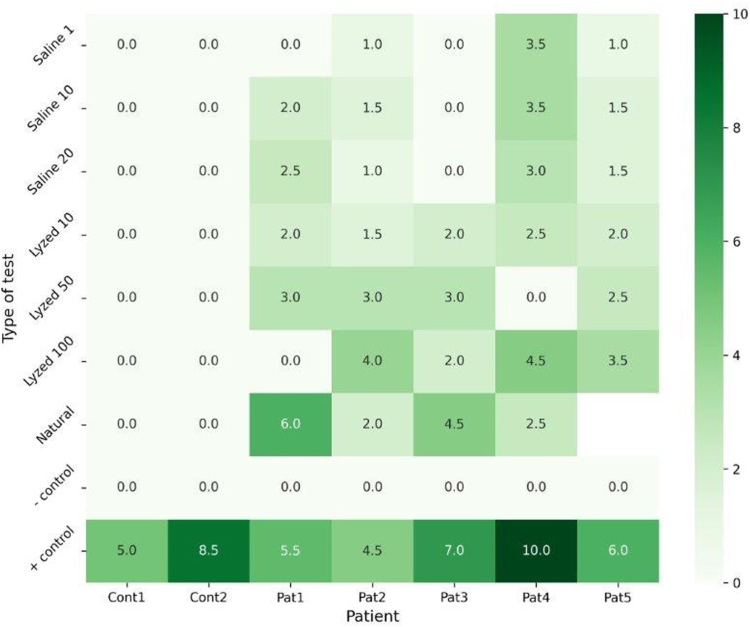
BR samples used in skin prick testing included spores in undiluted saline, a spore-free solution of soluble proteins (in 3 dilutions [1, 10, and 20 μg/mL]), and extracts from infected leaves (in 3 dilutions [10, 50, and 100 μg/mL]). Histamine served as the positive control, and sterile saline (0.9% NaCl) as the negative control. A minimum of 1 wheal with a diameter of at least 3 mm was considered a positive test result.

As no commercial sera test is available for analyzing specific IgE (s-IgE) to BR proteins, we extracted proteins from environmental samples. s-IgE reactivity against BR spores was identified in 10 of 13 patient sera (76.9%). In contrast, none of the 7 control sera exhibited IgE reactivity against BR. Moreover, all of the patients subjected to skin prick testing (SPT) (n = 5) elicited a wheal 3 mm or larger from at least 1 of the 7 variants of solutions containing BR, whereas the controls (n = 2) tested negative to all the solutions (Fig 3).

The findings of this pilot study suggest a possible association between BR spore dispersion and allergic airway symptoms. Susceptible individuals reported symptoms such as rhinitis, conjunctivitis, and/or asthma during the BR spore season, whereas the allergy-free controls experienced no such symptoms. Sensitization to BR spores, assessed by s-IgE testing and SPT, was observed among the patients but not among the controls. Although this study was limited by its small sample size (which reduced statistical power and generalizability), these findings provide preliminary evidence supporting BR as a clinically relevant seasonal allergen. As a proof of concept, this study also demonstrated the feasibility of combining daily symptom monitoring with BR spore exposure data and targeted sensitization testing, thus supporting its application in future larger-scale studies.

As the BR dispersal season coincides with a period of minimal activity of other seasonal aeroallergens,^11^ the likelihood that the observed symptoms are attributable to pollen or other fungal allergens is reduced. However, the patients had a higher burden of atopic diseases than the allergy-free controls, which may predispose them to nonspecific airway reactivity. To better isolate BR-specific effects, future studies should include symptom monitoring both during and outside the BR season. Individual BR exposure could not be measured directly, as spore counts were obtained from a regional monitoring station and may not accurately reflect personal exposure owing to variation in daily activities, locations, and microenvironments. Therefore, symptom-exposure associations were evaluated by comparing group-level spore data with mean patient symptom scores. This approach may have reduced the ability to detect a significant correlation between exposure and symptom severity. Nevertheless, a positive, albeit nonsignificant, association was observed, thus supporting the need for further investigation into the potential role of BR as an allergen.

Although sensitization to BR was demonstrated by using s-IgE testing and SPT, all analyses were conducted with nonstandardized extracts, as BR is not yet medically recognized as an allergen and no commercial diagnostics are available. This may have limited test sensitivity and specificity. Given that low protein concentrations are a known characteristic of fungal extracts,^16^^,^^17^^,^^19^^,^^20^ false-negative results remain the primary concern. However, our findings indicate IgE-binding reactivity to BR and support its potential as a clinically relevant allergen in susceptible individuals.

Shifts in the spectrum of airborne allergens, partly owing to human-induced climate change, highlight the need for refined exposome assessment to uncover novel interactions between environmental exposures, sensitization, and allergic disease.^21^^,^^22^ Our findings indicate that BR spores may function as aeroallergens and represent an underrecognized source of seasonal allergic airway symptoms in autumn. This is particularly relevant given the rising prevalence of allergic diseases and the expanding distribution of BR. Further studies are needed to confirm these preliminary observations as well as to better understand the underlying nature of BR allergy. This may help guide future diagnostic and monitoring strategies.

**Clinical implications**

Clinicians should consider BR spores as potential autumnal allergens in northern regions. Including BR in future diagnostic allergen panels may improve the identification and management of seasonal allergic airway diseases.

## Disclosure statement

Supported by the Northern Norway Regional Health Authority, the University Hospital of North Norway, the Norwegian Asthma and Allergy Association research fund, the Odd Berg Group medical research fund, and the Luxembourg Ministry of Higher Education and Research. None of the founding sources were involved in study design, collection, analysis, or interpretation of data; writing of the report; or decision to submit the article for publication.

Disclosure of potential conflict of interest: The authors declare that they have no relevant conflicts of interest.

## Figures and Tables

**Supplementary Figure S1:**
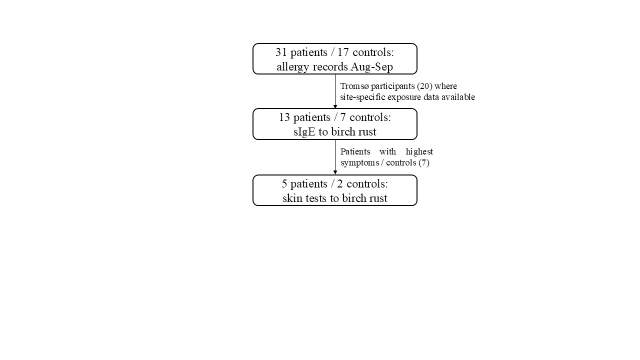


**Supplementary Figure S2:**
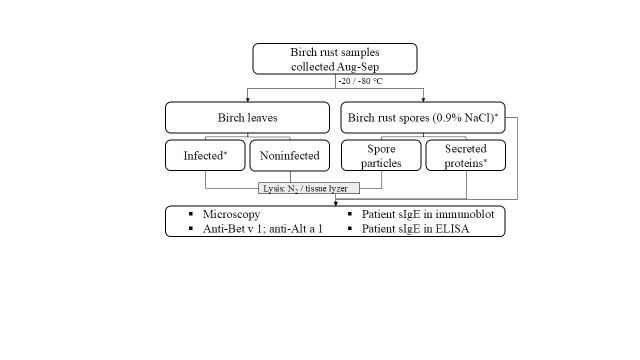


## References

[1] Genuneit J., Seibold A.M., Apfelbacher C.J., Konstantinou G.N., Koplin J.J., La Grutta S. (2017). Overview of systematic reviews in allergy epidemiology. Allergy.

[2] Dierick B.J.H., van der Molen T., Flokstra-de Blok B.M.J., Muraro A., Postma M.J., Kocks J.W.H., van Boven J.F.M. (2020). Burden and socioeconomics of asthma, allergic rhinitis, atopic dermatitis and food allergy. Expert Rev Pharmacoecon Outcomes Res.

[3] Jutel M., Agache I., Zemelka-Wiacek M., Akdis M., Chivato T., Del Giacco S. (2023). Nomenclature of allergic diseases and hypersensitivity reactions: adapted to modern needs: an EAACI position paper. Allergy.

[4] Haahtela T., Valovirta E., Saarinen K., Jantunen J., Lindström I., Kauppi P. (2021). The Finnish Allergy Program 2008-2018: society-wide proactive program for change of management to mitigate allergy burden. J Allergy Clin Immunol.

[5] Michaelis L.J., Skypala I.J., Gardner J., Sheikh A., Fox A.T., Holloway J.A. (2019). Upskilling healthcare professionals to manage clinical allergy. Clin Exp Allergy.

[6] Fauquert J.L., Alba-Linero C., Gherasim A., Testera-Montes A., Bentabol-Ramos G., Saenz de Santa Maria-Garcia R. (2023). Organ-specific allergen challenges in airway allergy: current utilities and future directions. Allergy.

[7] Nappi E., Paoletti G., Malvezzi L., Ferri S., Racca F., Messina M.R. (2022). Comorbid allergic rhinitis and asthma: important clinical considerations. Expert Rev Clin Immunol.

[8] Dramburg S., Hilger C., Santos A.F., de Las Vecillas L., Aalberse R.C., Acevedo N. (2023). EAACI Molecular Allergology User's Guide 2.0. Pediatr Allergy Immunol.

[9] Anees-Hill S., Douglas P., Pashley C.H., Hansell A., Marczylo E.L. (2022). A systematic review of outdoor airborne fungal spore seasonality across Europe and the implications for health. Sci Total Environ.

[10] Frisk C.A., Brobakk T.E., Ramfjord H. (2024). Allergenic pollen seasons and regional pollen calendars for Norway. Aerobiologia.

[11] Ramfjord H, Brobakk TE. Registrering av pollen og sporer. Astma og Allergiforbundet, NTNU, Institutt for biologi, Helsedirektoratet; 2020-2023. 100 p.

[12] Roy K.K. (2020).

[13] Bousquet J., Van Cauwenberge P., Khaltaev N. (2001). Allergic rhinitis and its impact on asthma. J Allergy Clin Immunol.

[14] Wise S.K., Lin S.Y., Toskala E., Orlandi R.R., Akdis C.A., Alt J.A. (2018). International consensus statement on allergy and rhinology: allergic rhinitis. Int Forum Allergy Rhinol.

[15] Seidman M.D., Gurgel R.K., Lin S.Y., Schwartz S.R., Baroody F.M., Bonner J.R. (2015). Clinical practice guideline: allergic rhinitis. Otolaryngol Head Neck Surg.

[16] Croce M.A., da Costa Manso E.R., Gambale W., Takayama L., Oliveira Andrade C.E., Pereira Pinto J.H. (2001). Sensitization to the fungus Hemileia vastatrix (coffee leaf rust). Allergy.

[17] Simon-Nobbe B., Denk U., Pöll V., Rid R., Breitenbach M. (2008). The spectrum of fungal allergy. Int Arch Allergy Immunol.

[18] Fakta om pollenallergi. https://www.naaf.no/allergi/pollenallergi/fakta-om-pollenallergi.%202025.

[19] Lopez M., Salvaggio J., Butcher B. (1976). Allergenicity and immunogenicity of Basidiomycetes. J Allergy Clin Immunol.

[20] Forkel S., Beutner C., Schröder S.S., Bader O., Gupta S., Fuchs T. (2021). Sensitization against fungi in patients with airway allergies over 20 years in Germany. Int Arch Allergy Immunol.

[21] Papadopoulos N.G., Agache I., Bavbek S., Bilo B.M., Braido F., Cardona V. (2012). Research needs in allergy: an EAACI position paper, in collaboration with EFA. Clin Transl Allergy.

[22] Pacheco S., Guidos-Fogelbach G., Pawankar R., D’Amato G., Latour-Staffeld P., Urrutia-Pereira M. (2021). Climate change and global issues in allergy and immunology. J Allergy Clin Immunol.

