# Supplementary material for: Birch rust allergy as a novel autumnal trigger of seasonal airway symptoms
Source: J Allergy Clin Immunol Glob. 2025 Nov 6;5(1):100599. doi: 10.1016/j.jacig.2025.100599 (PMC12670898; doi:10.1016/j.jacig.2025.100599)
Supplement: Supplementary Material [file mmc1.docx]

**Online Repository Methods**

**Study cohort**

In this cohort study, we enrolled 31 adult patients (mean age 45.1 y, SD 13.0) who reported symptoms of allergic conjunctivitis, rhinitis and/or allergic asthma annually from August to October (Figure E1). Participants with perennial symptoms without autumnal worsening were excluded. Inclusion was based on self-reported symptom history, as no validated diagnostic tools currently exist for BR allergy. Emphasis on subjective symptom reporting aligns with clinical practice, where allergy diagnosis typically relies on patient history supported by sensitization testing via specific IgE and/or skin prick testing (SPT)(1).

Seventeen non-allergic adults (mean age 44.2 y, SD 10.9) were recruited as controls. While not a randomized population sample, controls were deliberately recruited to ensure a clear clinical contrast and reduce the risk of misclassification. This approach also aimed to avoid inclusion of undiagnosed BR-sensitized individuals, which could confound group comparisons.

No formal power calculation was conducted, as this was a pilot study with an exploratory aim—both in evaluating BR as a potential seasonal allergen and in testing the feasibility of the study design and methods (2).

All participants were recruited at the outpatient clinic of the Regional Center for Asthma, Allergy, and Intolerance at the University Hospital of North Norway. The Regional Ethics Committee of Norway approved this study (Regional Committee for Medical and Health Research Ethics, REK N^o^62924), and written informed consent was obtained from all study participants. The study was registered in the ClinicalTrials.gov database (Identifier: NCT04322838).

**Clinical presentation during exposure period**

Participants recorded their allergic symptoms from August 4 to October 14, 2020. Every other day during this period, a Research Electronic Data Capture (REDCap) link was e-mailed to them. The link contained four questions on allergic airway symptoms, each graded using a Visual Analogue Scale (VAS). Separate VAS scores were provided for conjunctivitis, rhinitis, and asthma, along with an overall score for total allergy symptoms (see Symptom Registration Questionnaire below). Participants also reported current use of allergy and asthma medications (“yes”/ “no”) and specified the treatment used.

VAS was employed to assess symptoms, as it is a reliable and intuitive tool for capturing fluctuations in allergy severity. Due to its continuous format, VAS allows participants to indicate symptom intensity without being constrained by fixed categories, making it particularly well-suited for daily monitoring. No specific training was required, as VAS is generally considered simple to use and has been validated as both sensitive and reproducible in allergy research. Symptom intensity was interpreted using the MACVIA-ARIA–defined cut-off values for allergic rhinitis, where a VAS score >5 indicates uncontrolled symptoms, 2–5 indicates partly controlled symptoms, and <2 indicates controlled symptoms (3-5).

Supplementary data on previous and current allergies, asthma, and eczema were collected using a condensed version of the MeDALL questionnaire (Mechanisms of the Development of ALLergic Diseases; EU project FP7-CP-IP) (6). This version included 43 of the 88 questions from the original pediatric questionnaire, see MeDALL Questionnaire below. Participants completed the questionnaire once during the symptom registration period. Research Electronic Data Capture (REDCap) was used to distribute the questionnaire and collect responses from participants.

**Clinical definitions used in Table 1**

Current asthma (last 12 months): At least two of the following 3 criteria: 1) self-reported doctor-diagnosed asthma ever, 2) any indicative symptom in the last 12 months (wheezing, shortness of breath, dry cough at night), and 3) use of asthma medication in the past 12 months (7).

Current allergic rhinitis (last 12 months): At least two of the following 3 criteria: 1) self-reported doctor-diagnosed allergic rhinitis ever, 2) symptoms of sneezing, a runny or blocked nose, and 3) itchy, red and watery eyes after exposure to furred pets, pollen or house dust mite (8).

Current eczema (Last 12 months): At least two of the following 3 criteria: a) self-reported doctor-diagnosed eczema ever, b) dry skin, itchy rashes for two weeks or more in the past 12 months, and c) self-reported eczema combined with use of topical corticosteroids in the past 12 months (8).

**Environmental registration of birch rust spore counts**

Since 1980, there have been national registrations of daily pollen and spore content in the air, published annually in a report by The National Pollen Forecast for Norway. These registrations occur at 12 different stations across Norway, including one in Tromsø. Using pollen and spore traps designed for volumetric collection, these stations provide continuous recordings of pollen and spore dispersion throughout the season, typically spanning from March to September. Most particles collected in the traps originate from vegetation within a 10 km radius, although there is also a certain, yet unknown, number of particles transported from more distant locations. Characterization and counting of particles are carried out using microscopy (9).

BR has been included in these registrations since 2004, although it has not previously been considered a potential trigger for seasonal allergies. But the spores are morphologically distinct and readily identifiable in trap samples (9). With access to dispersal data, we were able to compare the spread of BR spores with allergy symptoms recorded by the participants. However, individual-level BR exposure data were not available, limiting the ability to directly correlate symptom trajectories with spore concentrations.

**Collecting birch rust samples for further analyses**

Birch leaves affected by BR display distinctive bright yellow spots. During August to September 2020, we gathered infected leaves from nearby birch forests in Tromsø. The birch rust fungi were then removed from the collected infected leaves using swabs soaked in sterile saline solution (0.9% NaCl) before being stored and frozen in plastic tubes with saline. Additionally, some contaminated infected birch leaves and some non-infected birch leaves were directly frozen for later protein extraction.

**Preparation of birch rust extracts**

Chemicals and reagents were from Sigma-Alderich (St. Louis, US) if not otherwise stated.

Both birch rust fungi and the non-/infected birch leaves were used for subsequent protein extraction and IgE binding assays (Figure E2).

Protein extraction. Samples of spores collected in saline were separated through filtration (Filter Paper Grade 595 ½, Whatman, Maidstone, UK) into a spore-free solution of soluble proteins and pellet of spores (Figure E2). Protein extracts were prepared from infected leaves (infected areas only), non-infected leaves and the spore pellet, as reported previously (10). Briefly, raw materials were grinded in liquid nitrogen to produce a fine powder. The powder was resuspended in lysis buffer (50 mM Tris-HCl, 150 mM NaCl, pH 8.0, 1% Triton X-100) and applied for protein extraction through plant tissue disruption (10 min, 30 Hz; Mixer Mill MM400, Retsch, Haan, Germany), followed by incubation (1 hr, 4°C; rotary shaker) and centrifugation (20,000 x g, 20 min, 4°C).

Protein quantification. The protein content of all samples, spores in saline, the spore-free solution of soluble proteins as well as the extracts from leaves and spores, were determined using the Bradford assay according to the manufacturers’ protocol (Protein assay dye 5000006, Bio-Rad, Winninglaan, Temse, Belgium), using a standard curve of known concentrations of bovine serum albumin (BSA).

Microscopy. To revise the presence or absence of intact spores, all samples were analyzed by microscopy (Inverted Microscope System Axio Observer Z1, AxioCam IC camera, Plan-Apochromat 63x/1, 40 Oil DIC M27 objective, Zeiss, Jena, Germany).

Protein separation and Western blot. Protein extracts were analysed by SDS-PAGE, followed by immunodetection using methods as described before (11, 12). In short, we revised the presence of the major birch pollen allergen (Bet v 1) and the major mold allergen (Alt a 1) using monoclonal mouse antibodies (1:10,000, Mab3B4 anti-Bet v 1, 1:10,000, Mab121 anti-Alt a 1, Indoor Biotech, Charlottesville, US). Human sera were 3- to 5-fold diluted in blocking buffer, 3% BSA, 0.3% Tween 20 in Tris-buffered saline (50 mM Tris, pH 7.4). Secondary antibodies were anti-mouse IgG-alkaline phosphatase (AP) conjugates (1:10,000, A2429, Sigma) and anti-human IgE-AP conjugates (1:500, 9160-04, Southern Biotechnology), followed by nitro-blue tetrazolium chloride/5-bromo-4-chloro-3'-indolyphosphate p-toluidine salt (NBT/BCIP) colorimetric revelation.

ELISA. Enzyme-linked immunosorbent assay (ELISA) was used to quantify sIgE in patient sera using a method reported earlier (10). Briefly, patient sera were 3- to 5-fold diluted in blocking buffer containing 3% bovine serum albumin (BSA). Sera pools of non-atopic individuals were used as negative controls, resulting in a 5-fold lower mean background than the cutoff value (0.1 kU_A_/L). Similar as to immunoblots, Bet v 1 and Alt a 1 were also tested in ELISA to review their presence in all extracts.

**IgE-analysis**

In a subgroup of 20 participants, consisting of 13 patients and 7 controls, we gathered data on serum total IgE and specific IgE (s-IgE) (Table 2). Common and recognized aeroallergens were assessed using the ImmunoCAP method (Phadia-Thermofisher, Upsala, Sweden). Inhalation panels 6 and 7 were employed for s-IgE screening, encompassing allergen extracts from pollens (grass, birch, mugwort), molds (*Cladosporium herbarum*, *Alternaria alternata*), house dust mite (HDM) and animal dander (cat, dog, horse, rabbit). Analysis of s-IgE to all specific allergens in the panel was conducted if the panel exceeded 0.1 kU_A_/L. Additionally, we tested s-IgE against the fungi Penicillium notatum, Mucor racemosus and Aspergillus fumigatus. Measurements of s-IgE against BR were carried out at Luxemburg Institute of Health (LiH) using extracts and methods described under *Preparation of birch rust extracts.*

**Skin prick test**

Data from skin prick test (SPT) were gathered from 5 patients and 2 controls and were conducted following standard procedures (13, 14). Birch rust samples used in the SPT included spores in undiluted saline, a spore-free solution of soluble proteins (in 3 dilutions: 1, 10, 20 µg/mL), and extracts from infected leaves (in 3 dilutions: 10, 50, 100 µg/mL). Histamine served as the positive control, and sterile saline (0.9% NaCl) as the negative control. Duplicate SPTs were performed, and wheal diameters were measured after 15 minutes. At least one wheal ≥ 3mm were considered as a positive result (14).

**Statistics**

The results primarily comprise descriptive data stratified by patients and controls. To assess the association between allergic symptoms and BR spore exposure, we used a mixed model multilevel linear regression with patient as the grouping level. The model was adjusted for anti-allergic and anti-asthmatic treatment, rhinitis in the last 12 months, asthma in the last 12 months, pollen allergy, perennial allergy, family history of atopy, and ever atopic disease. Statistical significance was set at a 5% level.

**References**

1. Roberts G, Pfaar O, Akdis CA, Ansotegui IJ, Durham SR, Gerth van Wijk R, et al. EAACI Guidelines on Allergen Immunotherapy: Allergic rhinoconjunctivitis. Allergy. 2018;73(4):765-98.

2. Thabane L, Ma J, Chu R, Cheng J, Ismaila A, Rios LP, et al. A tutorial on pilot studies: the what, why and how. BMC Medical Research Methodology. 2010;10(1):1.

3. Klimek L, Bergmann K-C, Biedermann T, Bousquet J, Hellings P, Jung K, et al. Visual analogue scales (VAS): Measuring instruments for the documentation of symptoms and therapy monitoring in cases of allergic rhinitis in everyday health care. Allergologie. 2018;41:364-74.

4. Bousquet P-J, Combescure C, Klossek J-M, Daurès J-P, Bousquet J. Change in visual analog scale score in a pragmatic randomized cluster trial of allergic rhinitis. Journal of Allergy and Clinical Immunology. 2009;123(6):1349-54.

5. Demoly P, Bousquet PJ, Mesbah K, Bousquet J, Devillier P. Visual analogue scale in patients treated for allergic rhinitis: an observational prospective study in primary care: asthma and rhinitis. Clin Exp Allergy. 2013;43(8):881-8.

6. Bousquet J, Anto JM, Akdis M, Auffray C, Keil T, Momas I, et al. Paving the way of systems biology and precision medicine in allergic diseases: the MeDALL success story: Mechanisms of the Development of ALLergy; EU FP7-CP-IP; Project No: 261357; 2010-2015. Allergy. 2016;71(11):1513-25.

7. Gough H, Grabenhenrich L, Reich A, Eckers N, Nitsche O, Schramm D, et al. Allergic multimorbidity of asthma, rhinitis and eczema over 20 years in the German birth cohort MAS. Pediatr Allergy Immunol. 2015;26(5):431-7.

8. Ballardini N, Bergström A, Wahlgren C-F, van Hage M, Hallner E, Kull I, et al. IgE antibodies in relation to prevalence and multimorbidity of eczema, asthma, and rhinitis from birth to adolescence. Allergy. 2016;71(3):342-9.

9. Ramfjord H, Brobakk TE. Registrering av pollen og sporer. Astma og Allergiforbundet, NTNU, Institutt for biologi,

Helsedirektoratet; 2020-2023.

10. Kuehn A, Hilger C, Lehners-Weber C, Codreanu-Morel F, Morisset M, Metz-Favre C, et al. Identification of enolases and aldolases as important fish allergens in cod, salmon and tuna: component resolved diagnosis using parvalbumin and the new allergens. Clin Exp Allergy. 2013;43(7):811-22.

11. Kalic T, Morel-Codreanu F, Radauer C, Ruethers T, Taki AC, Swoboda I, et al. Patients Allergic to Fish Tolerate Ray Based on the Low Allergenicity of Its Parvalbumin. J Allergy Clin Immunol Pract. 2019;7(2):500-8.e11.

12. Klueber J, Costa J, Randow S, Codreanu-Morel F, Verhoeckx K, Bindslev-Jensen C, et al. Homologous tropomyosins from vertebrate and invertebrate: Recombinant calibrator proteins in functional biological assays for tropomyosin allergenicity assessment of novel animal foods. Clin Exp Allergy. 2020;50(1):105-16.

13. Burbach GJ, Heinzerling LM, Edenharter G, Bachert C, Bindslev-Jensen C, Bonini S, et al. GA(2)LEN skin test study II: clinical relevance of inhalant allergen sensitizations in Europe. Allergy. 2009;64(10):1507-15.

14. Heinzerling L, Mari A, Bergmann KC, Bresciani M, Burbach G, Darsow U, et al. The skin prick test - European standards. Clin Transl Allergy. 2013;3(1):3.

**Symptom Registration Questionnaire**

To be sent via email for electronic completion every other day during two periods from August 4 to October 14, 2020.

You are asked to rate the allergic symptoms you experienced yesterday and today on a scale from 0 to 10, where 0 indicates no symptoms and 10 indicates severely disruptive symptoms:

1. Overall, how bothersome were your allergy symptoms YESTERDAY?
2. How bothersome were your nasal symptoms (e.g., sneezing, itching, runny nose, nasal congestion, forehead pressure/pain, throat mucus) YESTERDAY?
3. How bothersome were your eye symptoms (e.g., itching, redness, and tearing) YESTERDAY?
4. How bothersome were your asthma symptoms (e.g., chest tightness, coughing, wheezing, and shortness of breath) YESTERDAY?
5. Overall, how bothersome were your allergy symptoms TODAY?
6. How bothersome were your nasal symptoms (e.g., sneezing, itching, runny nose, nasal congestion, forehead pressure/pain, throat mucus) TODAY?
7. How bothersome were your eye symptoms (e.g., itching, redness, and tearing) TODAY?
8. How bothersome were your asthma symptoms (e.g., chest tightness, coughing, wheezing, and shortness of breath) TODAY?

**MeDALL Questionnaire**

Asthma and dyspnoe

**1) In the past 12 months, have you had wheezing or whistling in your chest?**
If no: go to question 5
If yes:

**2) How many times have you experienced these symptoms in the past 12 months?**

- 1-3
- 4-12
- More than 12

**3) In the past 12 months, have you had trouble sleeping or woken up due to wheezing or whistling in your chest?**

- No
- Less than once a week
- 1 or more times a week

**4) In the past 12 months, have you missed school/work due to wheezing or whistling in your chest?**

- No
- Yes

**5) In the past 12 months, have you experienced breathing difficulties (shortness of breath, chest tightness, wheezing or whistling in your chest)?**

- No
- Yes

**6) In the past 12 months, have you had wheezing or whistling in your chest, shortness of breath, or troublesome coughing in connection with any of the following?**

- No, I have not had symptoms with any of these
- Cold air or fog
- Pets
- Birch pollen
- Grass pollen
- Mugwort pollen
- Air pollutants, tobacco smoke, strong odors, cooking fumes
- Between August 1 and November 1

**7) In the past 12 months, have you had wheezing or whistling in your chest, shortness of breath, or troublesome coughing in connection with physical exertion?**

- No
- Yes

**8) In the past 12 months, have you used any medications for asthma or breathing difficulties?**

- No, go to question 14
- Yes

**9) Which medications for asthma or breathing difficulties have you used in the past 12 months?**

- Bronchodilator: Bricanyl, Ventoline, Airomir, or Buventol
- Pulmicort, Flutide, Becotide, Giona, Beclomet, AeroBec, Budesonide, Alvesco, Fluticasone, or Asmanex
- Symbicort, Seretide, Oxis, Serevent, Onbrez, or Striverdi
- Seretide, Symbicort, Flutiform, Inuxair, Serkep, Relvar, DuoResp, Airflusal, Bufomix, or Salmex
- Atrovent, Ipraxa, Spiriva, Eklira, Seebri, Incruse, Duaklir, Ultibro, or Anora
- Trimbow or Trelegy
- Singulair or Montelukast tablets

**10) If you use bronchodilator medication (Bricanyl, Ventoline, Airomir, or Buventol), how often do you use it in a typical week?**

- Less than 2 times per week
- 2 times or more per week

**11) In the past 12 months, have you taken corticosteroid tablets for asthma or breathing difficulties?**

- No
- Yes

**12) In the past 12 months, have you had a dry cough at night without having a cold at the same time?**

- No
- Yes

**13) Have you been troubled by coughing most of the time, for at least 3 months per year?**

- No
- Yes. > How many years?

**14) Have you been troubled by mucus from your chest most of the time, for at least 3 months per year?**

- No
- Yes. > How many years?

**15) If you have breathing difficulties or asthma, has it:**

- Limited you in schoolwork/work?
- Limited you in leisure activities?
- Limited you from being outdoors?

**Skin Issues and Eczema**

**16) In the past 12 months, have you had problems with dry skin?**

- No -> go to question 18
- Yes

**17) In the past 12 months, have you applied moisturizing cream/lotion because of dry skin?**

- No
- Yes, less than 1 month
- Yes, 1-6 months
- Yes, more than 6 months

**18) In the past 12 months, have you had itchy rashes?**

- No > go to question 23
- Yes

**19) How long do your itchy rashes usually last?**

- Less than 1 week
- 1-2 weeks
- More than 2 weeks

**20) In which period(s) of the year have you had itchy rashes in the past 12 months? (You may select multiple options)**

- January - July
- February - August
- March - September
- April - October
- May - November
- June - December

**21) Has the itchy rash disappeared completely at any point in the past 12 months?**

- No
- Yes

**22) In the past 12 months, have you had difficulty sleeping or woken up due to itchy rashes?**

- No
- Less than once a week
- 1 or more times a week

**23) In the past 12 months, have you had eczema?**

- No > go to question 27
- Yes

**24) How long have you had eczema in total during the past 12 months?**

- Less than 1 month
- 1-3 months
- 3-6 months

**25) In the past 12 months, have you applied corticosteroid cream because of eczema?**

- No
- Yes, less than 1 month
- Yes, 1-6 months
- Yes, more than 6 months

**26) If you have skin issues or eczema, has it:**

- Limited you in schoolwork/work?
- Limited you in leisure activities?
- Limited you from being outdoors?

**Nose or Eye Symptoms**

**27) In the past 12 months, have you had sneezing, itchy nose, runny nose, or nasal congestion for more than 4 days without having a cold at the same time?**

- No, go to question 30
- Yes

**28) How long have you had these symptoms without having a cold at the same time in the past 12 months?**

- Less than 1 month
- 1-3 months
- 3-6 months
- More than 6 months

**29) In the past 12 months, have these nasal symptoms occurred at the same time as itchy, watery eyes?**

- No
- Yes

**30) During which period(s) have you experienced sneezing, itchy nose, runny nose, or nasal congestion in the past 12 months? (You may select multiple options)**

- January - July
- February - August
- March - September
- April - October
- May - November
- June - December

**31) In the past 12 months, have you experienced nose or eye symptoms, without having a cold, when exposed to any of the following?**

- No, I have not had symptoms from any of these
- Pets
- Birch pollen
- Grass pollen
- Mugwort pollen
- Dust mites
- Mold
- During the period from August 1st to November 1st
- Hiking in the forest between August 1st and November 1st
- Other

**32) Have you had difficulty sleeping due to nasal symptoms in the past 12 months?**

- No
- Yes

**33) In the past 12 months, have you taken any medication for allergic rhinitis/hay fever?**

- No, go to question 35
- Yes

**34) What medications for allergic rhinitis/hay fever have you used in the past 12 months?**

- Eye drops: Livostin, Zaditen, Opatanol, Emadine, Spersallerg, Lomudal, or Lecrolyn
- Nasal spray: Rhinox, Otrivin, Dexyl, Zymelin, Otrivin comp, or Zycomb
  - Antihistamine nasal spray: Livostin
- Cortisone nasal spray: Avamys, Nasonex, Flutide nasal, Budesonide, Rhinocort, Fluticasone, Mometasone, or Nasacort
  - Cortisone + Antihistamine nasal spray: Dymista
- Antihistamine tablets: Zyrtec, Cetirizine, Aerius, Telfast, Xysal, Loratadine, Clarityn, Ebastine, or Kestine
  - Cortisone tablets: Prednisolone or Betapred
  - Cortisone injection(s)

**35) Have you had nasal congestion in the past 12 months?**

- No
- Less than 10 days
- 10 days – 12 weeks
- 12 weeks or more

**36) Have you had mucus at the back of your throat in the past 12 months?**

- No
- Less than 10 days
- 10 days – 12 weeks
- 12 weeks or more

**37) Have you had reduced sense of smell in the past 12 months?**

- No
- Less than 10 days
- 10 days – 12 weeks
- 12 weeks or more

**38) Have you experienced pain or pressure around your forehead, nose, or eyes in the past 12 months?**

- No
- Less than 10 days
- 10 days – 12 weeks
- More than 12 weeks

**39) During which period in the past 12 months have you had symptoms of mucus at the back of your throat, reduced sense of smell, or pain/pressure around your forehead, nose, or eyes?** (You can select multiple options)

- January - July
- February - August
- March - September
- April - October
- May - November
- June - December

**40) If you have nasal or eye symptoms, have they:**

- Prevented you from doing schoolwork/work?
- Prevented you from participating in leisure activities?
- Prevented you from being outdoors?

**Family and doctor-diagnosed atopic diseases**

**41) Does anyone in your family (parents, siblings, children, or grandchildren) currently have or have had any of the following?**

- No, none of them
- Asthma
- Eczema
- Hay fever/respiratory allergies
- Food allergies

**42) Have you ever been diagnosed by a doctor with any of these conditions?**

- No, none of them
- Asthma
- Eczema
- Hay fever/respiratory allergies
- Food allergies

**43) Did you have any of these conditions as a child or adolescent?**

- No, none of them
- Asthma
- Eczema
- Hay fever/respiratory allergies
- Food allergies

**Figure legends:**

**Figure E1:** Organizational flowchart of the clinical pilot study on allergy to birch rust. Numbers in brackets represent the numbers of subjects for each step. Aug, August; Sep, September. sIgE, specific IgE.

**Figure E1:** Organizational flowchart of the clinical pilot study on allergy to birch rust. Numbers in brackets represent the numbers of subjects for each step. Aug, August; Sep, September. sIgE, specific IgE.
